# Using large and complex datasets for small-area environment-health studies: from theory to practice

**DOI:** 10.1093/ije/dyaa018

**Published:** 2020-04-15

**Authors:** Frédéric B Piel, Samantha Cockings

**Affiliations:** d1 UK Small Area Health Statistics Unit (SAHSU), Department of Epidemiology & Biostatistics, School of Public Health, Imperial College London, London, UK; d2 National Institute for Health Research Health Protection Research Unit (NIHR HPRU) in Health Impact of Environmental Hazards, Imperial College London, UK; d3 Department of Geography and Environmental Science, University of Southampton, Southampton, UK

Humans are exposed to a wide range of pollutants throughout their lifetime, many of which pose a potential risk to their health. Such hazards include features of the natural, human-modified, social and economic environments. In this supplement, we are primarily concerned with risks to human health resulting from hazards of the human-modified environment, although many of the concepts, methods and tools are equally applicable to investigations of the health impacts of other types of environmental hazards. Amongst human-modified environmental hazards, air pollution has been identified as the world’s largest killer, being responsible for an estimated 6.4 million deaths per year (1 in 9 deaths).^1^ According to the World Health Organization, two billion children live in areas where outdoor air pollution exceeds recommended international limits and 300 million children live in areas where outdoor air pollution exceeds six times those international limits. Other hazards of the human-modified environment include water pollutants, such as chemicals and microplastics; radiation from mobile phones, powerlines or nearby nuclear installations; and soil contaminants such as heavy metals.

When health risks are very high and localized, they tend to be rapidly identified by alert clinicians, public health professionals or members of the public. This can for example set the launch of a cluster investigation for which Public Health England recently published guidance.^2^ It is much harder to identify risks when they are less obvious and more ubiquitous. Small-area methods provide a powerful means to study health effects at local, regional or national level taking into account spatial heterogeneities in socio-demographic characteristics and environmental exposures.

In the UK, the UK Small Area Health Statistics Unit (SAHSU, www.sahsu.org) was established in 1987 to investigate the potential health effects of environmental pollutants, following reports of excess risks of leukaemia and non-Hodgkin lymphomas in young people living near the Sellafield nuclear plant.^3^ In recent years, SAHSU has undertaken a series of small-area studies to investigate *inter alia* potential health risks associated with emissions from municipal waste incinerators,^4–6^ air pollution,^7^ aircraft noise,^8^ disinfection by-products^9^ and exposure to non-ionizing radiation from living near mobile phone base stations or overhead powerlines.^10–12^

Although small-area studies have been used for a long time, their popularity seems to be increasing due to efforts to quantify local burden of diseases and risk factors. It is therefore timely to provide an overview of the strengths and limitations of this particular study design, and of recent methodological advances.

Building on over 30 years of expertise in SAHSU, the *Education Corner* manuscript accompanying this supplement^13^ presents an overview of the methodological steps and challenges associated with designing and completing small-area studies, including data access, data linkage and data privacy. The supplement itself provides a more detailed critique into the accessibility of small-area data and dissemination of results;^14^ novel methods to produce high-resolution population data;^15^ recent developments of statistical models to monitor non-communicable disease in both space and time;^16^ user-friendly tools to trace residential history when assigning environmental exposures,^17^ mapping disease risk and conducting risk analysis;^18^ and a practical example of a national small-area study investigating a specific source of exposure.^19^

Hodgson *et al*.^14^ provide a detailed description of the challenges involved in accessing, analysing and disseminating small-area data. Substantive changes to data access regulations pose significant challenges, given that the size of geographical units used in small-area studies often necessitates the use of sensitive data. Growing concerns over data privacy and confidentiality, reflected both by the implementation of the EU General Data Protection Regulation (GDPR) in May 2018 and the launch of the National Data Opt-out Programme on the same day in the UK, mean that requirements to access sensitive data are becoming stricter over time. In parallel, developing studies in collaboration with members of the public and representatives of various stakeholders—or co-design—is becoming more common. Although both of these are welcome developments, they need to be carefully considered during the development and implementation of any small-area study.

Using examples from the UK and the US, Fecht *et al.*^15^ describe novel methods to produce time-specific high resolution denominator data for small-area studies by combining new and emerging forms of data (such as sensed footfall or traffic data) with traditional data sources (such as census or surveys). They outline the challenges involved in using new data sources such as the American Community Survey to produce estimates of population at risk and describe openly available software (e.g. SurfaceBuilder247) that facilitates the creation of gridded population distribution models for specific times and dates.

Blangiardo *et al.*^16^ present a review of recent advances in spatio-temporal disease surveillance for non-communicable diseases (NCDs) using Bayesian hierarchical methods. They discuss key challenges in dealing with NCD surveillance, particularly how to account for false detection and the modifiable areal unit problem. Traditional models focused on identifying either spatial or temporal patterns, whereas more recent methods, such as the hierarchical models described, enable dependencies between data sources to be exploited in both space and time. Furthermore, the Bayesian framework allows uncertainties to be quantified and taken into account throughout the modelling approach.

Public health authorities often need to provide rapid assessment of potential health risks in a given area. This may follow e.g. the report of a suspected cluster of disease cases. Piel *et al.*^18^ describe the functionalities of the latest version of the Rapid Inquiry Facility (RIF) that has been developed by SAHSU as an open access software for disease mapping and risk analysis at small-area level.

Estimation of exposure to environmental pollutants often depends on information on residential address. Accurate reconstruction of residential histories can greatly reduce bias in exposure assignment in such studies, particularly in those spanning long time periods. Fecht *et al.*^17^ present an algorithm for undertaking this complex and time-consuming task. They illustrate its use in constructing prenatal and early-life air pollution exposure for 14 541 pregnant women participating in the Avon Longitudinal Study of Parents and Children (ALSPAC) in the South West of England.

The final paper in the supplement, a study by Toledano *et al.*,^19^ investigates the hypothesis that air ion density or electric fields in the vicinity of high-voltage overhead power lines may be associated with cancer risk in adults.

We hope that the expertise shared in this supplement and the examples provided highlight the ongoing usefulness of the small-area study design and will help readers conduct their own rigorous small-area studies to assess the health impacts of environmental hazards.
